# The role of prefrontal cortex in cognitive control and executive function

**DOI:** 10.1038/s41386-021-01132-0

**Published:** 2021-08-18

**Authors:** Naomi P. Friedman, Trevor W. Robbins

**Affiliations:** 1 Department of Psychology & Neuroscience and Institute for Behavioral Genetics, University of Colorado Boulder, Boulder, CO USA; 2 Department of Psychology and Behavioural and Clinical Neuroscience Institute, University of Cambridge, Cambridge, UK

**Keywords:** Cognitive control, Human behaviour, Psychiatric disorders

## Abstract

Concepts of cognitive control (CC) and executive function (EF) are defined in terms of their relationships with goal-directed behavior versus habits and controlled versus automatic processing, and related to the functions of the prefrontal cortex (PFC) and related regions and networks. A psychometric approach shows unity and diversity in CC constructs, with 3 components in the most commonly studied constructs: general or common CC and components specific to mental set shifting and working memory updating. These constructs are considered against the cellular and systems neurobiology of PFC and what is known of its functional neuroanatomical or network organization based on lesioning, neurochemical, and neuroimaging approaches across species. CC is also considered in the context of motivation, as “cool” and “hot” forms. Its Common CC component is shown to be distinct from general intelligence (*g*) and closely related to response inhibition. Impairments in CC are considered as possible causes of psychiatric symptoms and consequences of disorders. The relationships of CC with the general factor of psychopathology (p) and dimensional constructs such as impulsivity in large scale developmental and adult populations are considered, as well as implications for genetic studies and RDoC approaches to psychiatric classification.

## Introduction

Many psychiatric disorders and neurological conditions are associated with deficits in cognitive control (CC) and/or dysfunction of the prefrontal cortex (PFC) and its associated circuitry [1–4]. Consequently, there is a considerable premium on elucidating the basic psychological and neuronal mechanisms underlying the PFC’s role within the neural networks that regulate behavior and cognition.

CC is a term usually associated with the healthy functioning of the PFC and related regions such as the cingulate cortex [5]. Deriving from a cybernetic and cognitive neuroscience perspective, CC has often been considered synonymous with the earlier notion of executive function (EF), which has its roots in studies of clinical neuropsychology. In both cases, a core process of behavioral regulation is envisaged that optimises goal-directed behavior and counters automaticity. This process has many similarities with the distinction between controlled and automatic responding [6], which approximately aligns with the learning theory distinction between goal-directed and habitual responding [7]. One would expect the absence of CC to produce automatic behavior; controlled responding is goal-directed and flexible.

**Fig. 1:**
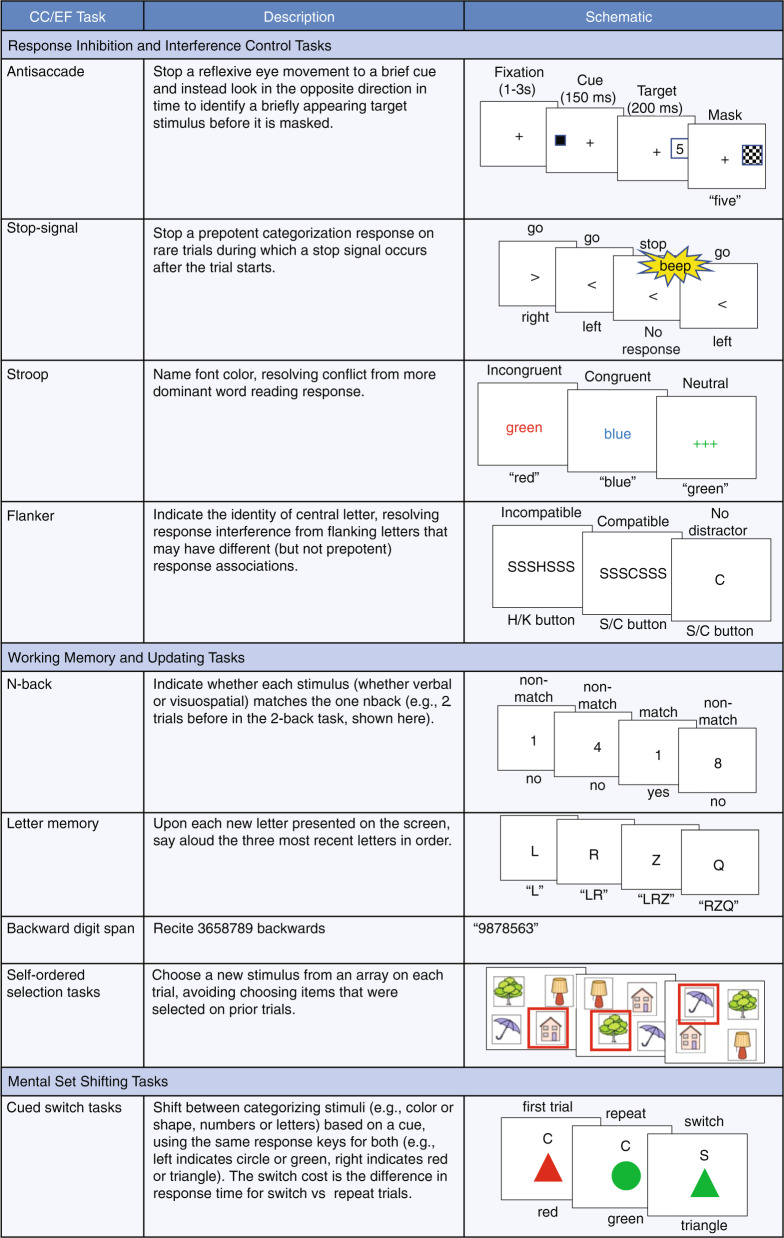

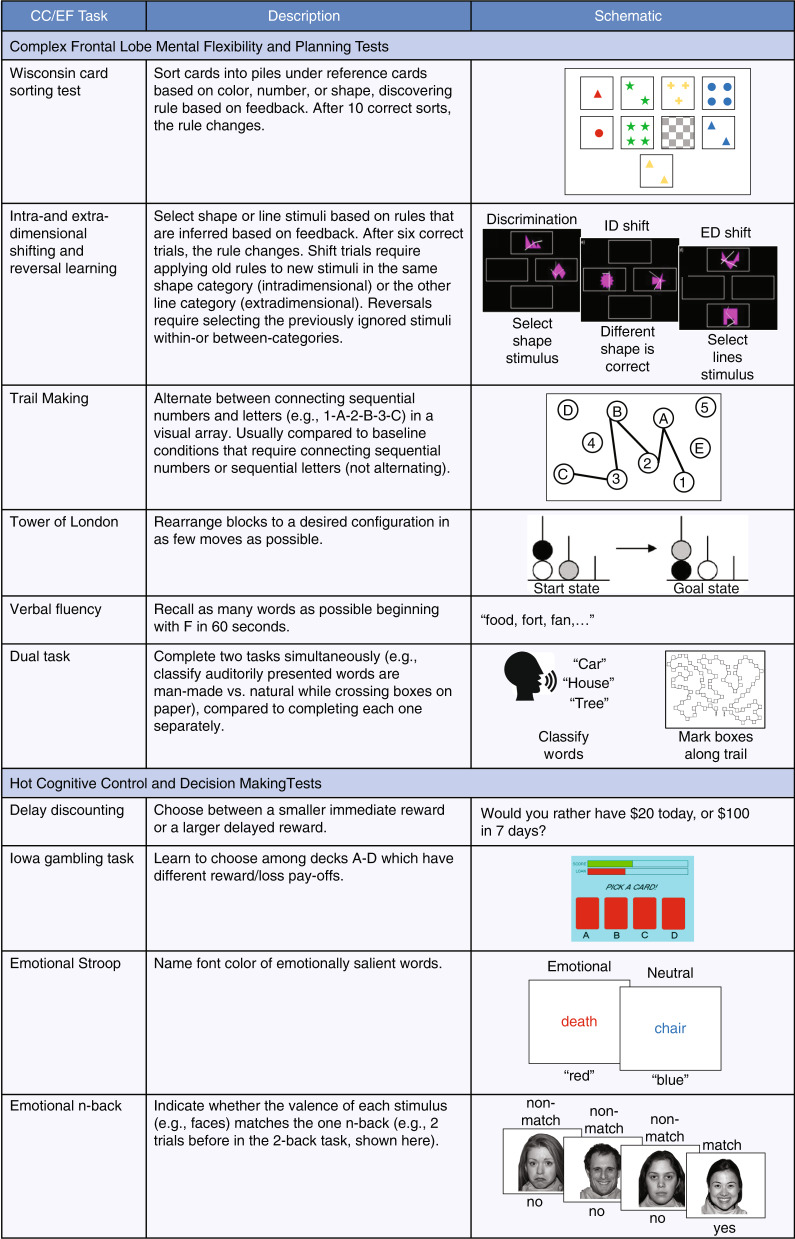
Commonly used cognitive control (CC)/executive function (EF) tasks. When relevant, text above each schematic indicates different conditions, and text below indicates correct responses. The faces included in the emotional *n*-back illustration are taken from the NimStim set of models who have granted permission to publish their images in scientific journals [246].

Miller and Cohen (2001) [8] proposed that “[CC] stems from the active maintenance of patterns of activity in the PFC that represent goals and the means to achieve them. They provide bias signals to other brain structures whose net effect is to guide the flow of activity along neural pathways that establish the proper mappings between inputs, internal states, and outputs needed to perform a given task” (p. 167). This seminal account of the role of PFC in CC essentially consists of the contextual biasing of attention (for example, via instructions) to resolve conflicts and exert attentional control. The typical example is the much-used Stroop interference paradigm (see Fig. 1), in which participants are required to name the color (e.g., green) of the ink used to print words whose meaning is incongruent with that color (e.g, RED). The greater pre-potency of reading words over-reporting color causes interference, manifested as a slowing of decisional latency and activation of the anterior cingulate cortex (ACC) [9]. The conflict can be resolved by focusing attention on the color of the ink, associated with control (or bias) exerted by PFC regions. The theory was supported by an fMRI study showing that ACC activation was accompanied by activations of the dorsolateral (dl)PFC associated with top-down adjustments of response control [10]. Hence, in the simple model proposed by Miller and Cohen [8], the ACC detects conflict that is resolved by top-down biasing of response options from the dlPFC [9]. This theoretical scheme has provided one of the first demonstrations of a CC process to be mediated by specific, interactive PFC circuitry.

One question to be considered here is whether CC is best considered as a unitary construct, a set of component processes, or a hybrid product of these extremes. If CC is best characterized as a set of distinct component processes, another question is how many of these can be identified and can they be further defined? The possible fractionation of CC can be related broadly to the heterogeneous nature of the PFC itself, which comprises, across species, many distinctive sub-regions, characterized by their cytoarchitectonic characteristics and by their connectivity with other brain regions. A related question then arises as to whether the PFC’s role in CC is that of a unitary entity or “multiple demand” (MD) system [11]. The MD system appeals to the enormous neural plasticity shown to be inherent within the PFC, so that the same neuronal ensembles can be recruited for superficially different tasks using “adaptive coding” [12–14]. However, an alternative viewpoint would be that the PFC sub-regions have somewhat different functions, potentially mediating the specific domains of CC. A more sophisticated version conceives specific PFC subregions of having multiple functions, as a consequence of the network-like nature of brain organization that has been revealed by brain imaging (see Haber et al., this issue, and Menon & D’Esposito, this issue [15, 16]). Yet a further view would argue that CC is emergent from network processing in the brain, and there is no particular network area that mediates control (see [17, 18]).

This article will consider these fundamental questions, beginning with the key issues of how CC is measured and how its unity and possible diversity have been evaluated at both the behavioral and neurobiological levels. In considering the neural substrates of CC, we draw upon the human neuropsychological and neuroimaging literature, as well as basic neuroscience studies in experimental animals. Clearly, these studies are well poised to address the question of dissociation of component processes, for example, via interventional techniques including lesions and neuropharmacological manipulations. A particular issue is how CC operates in different states of the internal or external environment, for example, following stress, that may alter the neurochemical ambience and functioning of the PFC and produce curvilinear, “inverted U-shaped,” functions of performance efficacy [19]. CC or EF in the past has also been related to other unitary constructs such as general intelligence, or *g* [11]. To what extent, therefore, are these entities the same, also entailing presumably similar neural substrates? In fact, we will discuss evidence that although these unitary neurocognitive constructs overlap to some extent, they are different.

Finally, we will consider clinical implications, particularly how CC or EF functions relate to personality traits or dimensions relevant to psychopathology, such as impulsivity and compulsivity, as well as the general psychopathological factor *p*, which captures covariance across a range of mental health disorders [20]. We will conclude with future research priorities, with an ultimate aim of determining how CC can be optimized for dealing with behavioral problems and mental health disorders.

## Psychological organization of CC

**Fig. 2:**
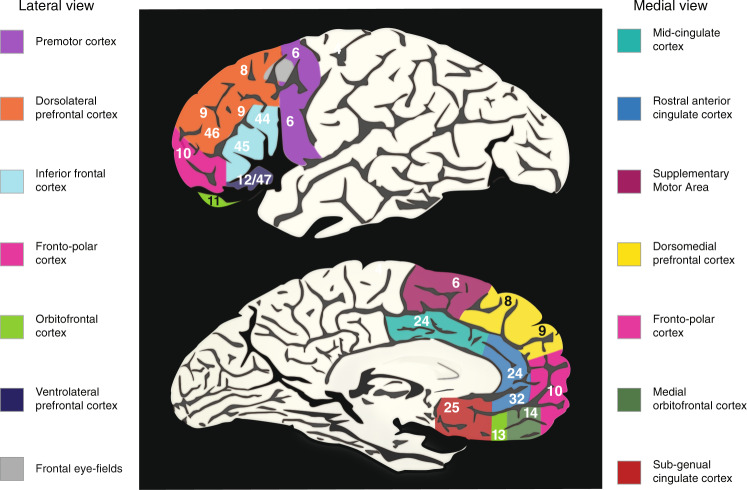
Major areas of the prefrontal cortex. The top panel depicts a lateral view, and the bottom panel depicts a medial view. Numbers indicate Brodmann Areas (BA). Note that the commonly described “ventromedial prefrontal cortex” potentially subsumes several BAs: 25, 32, 14, and possibly 11 and 13.

The neuroanatomical connectivity of the PFC to most parts of the cortical and subcortical brain makes it well suited for participating in a number of neural networks and carrying out CC operations in different functional domains (e.g., spatial, visual, and verbal). Moreover, PFC functions probably depend on specializations of dendritic branching and spine density of pyramidal cells, especially in the cycloarchitectonically distinct regions of the granular PFC (Fig. 2) [21–23]. The cellular physiology of these regions is characterized by rapid firing and properties of neural plasticity that may enable such functions as goal maintenance in working memory and the flexible functioning of an MD network [24–27].

However, it is a major challenge to deduce how the PFC is organized to mediate the range of cognitive processes referred to as CC/EF, which include stopping automatic or dominant responses, controlling interference, switching between tasks, coordinating multiple tasks, updating working memory, monitoring, and planning [28–30] (see Fig. 1 for illustrations of some example tasks used to assess these cognitive processes). The term “unity and diversity” was first used in 1972 to describe the relationships among such diverse frontal lobe processes: Teuber [31] observed a “bewildering variety in man’s reaction even to fairly restricted and non-progressive [prefrontal] lesions” (p. 637), that nevertheless, could be described broadly in terms of levels of “compulsiveness” or “abnormally stimulus-bound behavior” (p. 640). Similarly, Duncan and colleagues [32] reiterated this unity and diversity term to describe frontal lobe deficits after head injury: They observed uniformly low correlations among frontal lobe tests, yet a common element of “goal neglect, or disregard of a known task requirement” (p. 713).

**Fig. 3:**
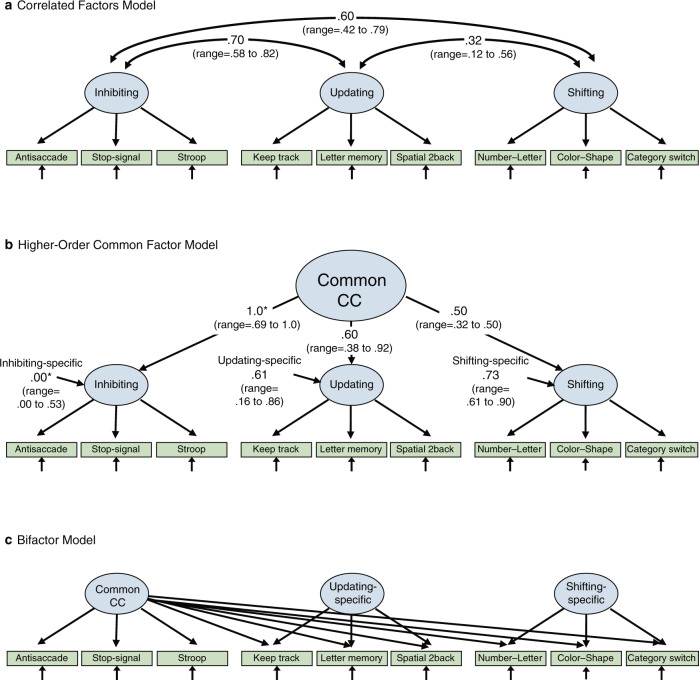
Latent factor models of cognitive control (CC). Proposed CC functions are represented as latent variables (depicted with ellipses) that predict variation in performance on specific tasks (rectangles) chosen to measure those abilities. Factor loadings are depicted with single-headed arrows between the factors and nine measured tasks. The short arrows indicate residual variances, the unique variance in each task that is unrelated to the CC factors, attributable to measurement error as well as reliable task-specific variation. **a** In a correlated factors model, tasks are predicted by CC factors that are allowed to be correlated, and unity and diversity are represented in the correlations between factors (represented with curved double-headed arrows). The numbers shown are the average correlations and the range of correlations from six studies using a similar battery (*N*s = 137–786). **b** A higher-order “Common” CC factor can also be used to model the correlations among the factors [39, 158]. This higher-order factor predicts the lower-order factors, and they correlate to the extent to which they are jointly predicted by the common factor. In such models, the diversity is captured by the residuals of these factors after the variance due to the common factor is removed (inhibiting-specific, updating-specific, and shifting-specific variances). The numbers shown indicate the average and range of factor loadings for the Common CC factor, and the corresponding averages and ranges for the residual variances for inhibiting, updating, and shifting factors (i.e., the variance not explained by the common factor), derived from the correlations in panel **a**. *indicates the standardized loadings were bound at 1, and the residual variances bound at zero. **c** Alternative model structures (called nested factors models or bifactor models) can be used to capture unity and diversity factors more directly. In these models, all tasks load on a common factor, but also load on orthogonal specific factors. These models thus partition each factor into variance that is common across all tasks and variance that is unique to tasks assessing particular processes. Although these alternative parameterizations typically do not result in appreciably different fits to the data, they can make it more convenient to examine relationships to other constructs of interest: Because the unity and diversity components are represented with orthogonal latent variables rather than in the correlations between factors or with residual variances, it is straightforward to discern whether a construct is related to the unity vs. diversity components.

The term “unity and diversity” has also been used to describe the pattern of correlations among laboratory CC tests in individuals without brain lesions. Specifically, Miyake et al. [33] investigated the structure of CC in college students by administering a battery of tasks, each designed to tap one of three CC abilities: inhibiting a prepotent response (stopping an automatic response, sometimes in order to make an alternative response), updating working memory (continuously replacing no-longer relevant information in working memory with newly relevant information when it is detected in the environment), or shifting between mental sets (switching between two alternative tasks). They administered multiple tasks to assess each of these three functions so that they could estimate a latent variable (a statistical extraction of the common variance in a set of tasks) for each function. They then examined the relationships among these functions at the level of these latent variables, rather than at the level of individual tasks. The basic model they examined is shown in Fig. 3a, along with alternative models examined in later studies (Fig. 3b, c).

This approach was motivated by the recognition that the low correlations observed in prior studies might reflect “task impurity” of CC measures. That is, CC is by definition control of other cognitive processes, and so performance in CC tasks may reflect variation in those other processes as well as the CC process of interest. Evaluating a CC ability in multiple contexts and statistically extracting what is common enables purer measures that are also free of random measurement error. Indeed, Miyake et al. [33] found that although the individual tasks showed relatively low correlations (*r* = –0.05 to 0.34), consistent with much prior research, the latent variable correlations were stronger (*r* = 0.42–0.63). These correlations were all significantly greater than zero, indicating that these three CC processes indeed shared something in common (they showed some “unity”). However, these three factors were also somewhat separable (they also showed some “diversity”): A model in which these nine tasks were explained by three correlated factors was superior to models that used fewer factors. Since that initial study, this psychometric latent variable approach has been used in a large number of studies to show that unity and diversity of CC is evident across samples and ages (e.g., [34–41]), although there are some studies that suggest more unity (higher correlations between factors) in early childhood [35, 42–44].

Although latent variable studies often focus on the same three constructs selected by Miyake et al. [33], their model was never intended to be comprehensive. Other candidate components of CC and taxonomies are considered in Box 1. Miyake et al. also recognized that commonly examined CC processes might comprise multiple components. For example, measures of working memory capacity and updating can include separable sub-functions like maintenance and removal of items in working memory [45]. Mental set-shifting tasks may require multiple sub-processes, including interference control, retrieval of task sets, and task-set reconfiguration [46]. And these intermediate levels may be combined with other functions (e.g., sequencing subgoals) to result in more complex postulated CC functions like planning [33].

Finally, goal-directed behavior or associated outcomes have motivational and value-based elements in decision-making cognition that raise the issue of whether CC can be distinguished from motivational control and value-based processes. Thus, for example, Koechlin [47] firmly distinguishes between CC and “motivational control.” Most models of CC focus on so-called “cool” tasks that use non-emotional stimuli, such as the color-word Stroop task or the *n*-back task with letters or neutral words. “Hot” CC can be measured with similar paradigms but using emotional stimuli (Fig. 1), thus assessing control over motivational or emotional information. For example, hot Stroop tasks might require naming the font color of emotionally salient words (e.g., “failure”) [48] or categorizing the emotional valence of words (e.g., “miserable”) in the context of faces with emotional expressions that conflict with those words (e.g., [49]). Hot CC can also be measured with tasks that do not have a cool analogue, such as gambling tasks and delay of gratification tasks [50, 51] (Fig. 1). Research with children suggests separability of hot and cool CC in terms of their relations with each other and with other measures [50, 52–57]. However, neuroimaging studies comparing CC tasks with non-emotional and emotional stimuli find that they involve similar CC regions [58, 59] (dorsal ACC, anterior insula, and lateral and medial PFC), but tasks with emotional conflict also recruit distinct neural regions related to salience and emotional processing (amygdala, more rostral areas of the ACC and medial PFC, and orbitofrontal cortex) [60–66]. Such patterns suggest that there may be common CC processes across hot and cool tasks.

Box 1 Alternative candidate components (or taxonomies) of CC Miyake et al.’s [33] model recognized that there could be other potential, separable CC constructs, besides the traditional triad of working memory, cognitive flexibility, and inhibition. How other candidate processes such as (attentional) monitoring; dual-tasking; strategic retrieval and generativity (including e.g., verbal fluency and episodic memory) [233, 247, 248]; “compositionality” [70]; self-report (e.g., impulsiveness); and metacognition [249] including social aspects (e.g., “theory of mind”), might relate to or derive from the original triad is unclear. Studies that have tested the relations of some of these candidate CC components have found that they are correlated with the more commonly examined CC processes but also show some separability [233, 247, 248]. Thus, the notion of unity and diversity is likely to apply to models that include more than the most typically examined three constructs.

One rough parcellation of alternative CC components has been achieved by anatomical localization in a large number of patients with frontal injuries [250–252]. The tasks included requirements to attend, switch, be vigilant, tap rhythmically, and respond quickly. Superior medial deficits were associated with “energization” (initiating and sustaining a response, problems with which were related to slower reaction times). Right lateral lesions were associated with “monitoring” (checking performance and adjusting behavior when necessary). Left lateral lesions were associated with “task setting” (setting up a stimulus-response relationship and organizing the processes necessary to complete a task), especially their acquisition and flexible use; and, for the inferior medial group, a problem of *maintaining* task set (possibly related to distractibility). These proposed CC components have only an approximate relationship with those discussed earlier (typically examined in latent variable models), and intriguingly do not appeal at all to any construct of “inhibition.” Rather, Stuss and Alexander [250] proposed that inhibition emerged as a combination of these three processes.

## Fractionation and integration of CC within PFC

The main methodologies employed for examining how PFC mediates CC have been (i) the anatomical localization of specific aspects of CC/EF, based for example on evidence of lesions in conjunction with correlative neurophysiological or neuroimaging methods; and (ii) the analysis of task performance using the mapping of neural network methodology aimed at elucidating the sequencing and overall integration of CC processes. With respect to the latter, resting state and functional connectivity data suggest several distinct configurations (networks) of PFC and other brain regions, reviewed by Menon and D’Esposito (this issue) [16]: the lateral “fronto-parietal” (or “central executive”) network (FPN), anchored in the dorsolateral (dl) and dorsomedial PFC and posterior parietal cortex; the “cingulo-opercular” network (CON), which overlaps with a “salience” network and includes the ACC, the insula, and subcortical regions; the “ventral attention” network, which includes inferior frontal gyrus, regions of the insula, and the temporoparietal junction; the “dorsal attention” network, which includes the frontal eye fields and intraparietal sulcus; and the “default mode network” (DMN) comprising medial PFC regions interacting with certain posterior cortical regions (Haber et al., this issue; Menon & d’Esposito, this issue [15, 16]). The DMN typically shows inverse levels of activity in relation to the other networks during external task performance, with the DMN being more active at rest, and consequently associated with “internal” control processes (Menon & D’Esposito, this issue [16]).

With respect to the neurobiological substrates of unity and diversity, one view would emphasize the participation of the PFC as a hub of an MD network that mediates all of the common facets of CC. Another would point to the cytoarchitectonic heterogeneity of the PFC (Fig. 2) and ask how the various components of CC were coordinated and integrated in different tasks by different PFC regions, and their specific roles as functional nodes within networks. The relevant circuitries include connectivity of the PFC to posterior cortical regions such as the parietal lobes and to subcortical regions, such as the striatum. Hybrid models of organization may incorporate MD characteristics in certain PFC regions, but also allow for specificity of neural connectivity, to mediate, for example, specific aspects of response inhibition, updating, or cognitive flexibility, as well as other forms of CC that putatively involve, for example, interactions with language systems and autobiographical episodic memory. An important consideration is the extent to which hierarchical CC processing maps onto a hierarchical lateral PFC system and how motivational processes interact with it to achieve integration of these dual forms of control [67]. The following sections selectively survey findings from the enormous literature on these issues (see also reviews by Tanji & Hoshi [68] and Badre [69]).

### Fractionation of CC

Evidence from the double dissociation of component CC processes is relevant to the question of whether PFC’s role in CC is unitary. By the time of the classic edited book, *The Frontal Granular Cortex and Behavior* [70], lesion studies in non-human primates had already shown, via the double dissociation strategy, considerable apparent localization of function within the frontal lobes. For example, whereas impairments in working memory test paradigms such as spatial delayed response were produced by lesions of the sulcus principalis (dlPFC), damage to more ventrolateral and orbitofrontal regions produced impairments in tests apparently measuring inhibition and cognitive flexibility, such as reversal learning and Go/No-Go responding. These findings apparently provided strong evidence against a unitary system.

In the case of *working memory*, the lateral frontal cortex in primates, already implicated in the spatial delayed response task, was known to contain cells that exhibited activity in delay periods [24] in response to stimuli in a number of sensory modalities. Goldman-Rakic [71] in particular suggested that the dlPFC (BA-46 /sulcus principalis) mediated the maintenance of spatial information in memory in preparation for action. Subsequent work queried whether the maintenance of information per se was a critical PFC function. Thus, it was shown from other electrophysiological evidence as well as human functional imaging that the anterior inferotemporal/perirhinal and parietal cortex also exhibited maintenance operations [72], although the dlPFC did appear to have important roles in resisting interference (e.g., distraction) in working memory [73]. Moreover, other investigators (e.g., [74]) have interpreted the role of the “mid-dorsal” (BA-9/46) PFC to mediate CC processes, such as the monitoring or tagging of recently selected items, as in self-ordered or *n*-back tasks, rather than the passive maintenance of information (see also [75] for a meta-analysis). By contrast, damage to a different sector of more posterior dlPFC (BA-8) impaired selection of motor responses to particular stimuli (conditional learning of task sets) but such performance was not affected by BA-9/46 damage. This type of double dissociation further supports the hypothesis that distinct processes of CC in lateral PFC are mediated by different regions and is relevant to hierarchical theories of CC, considered below.

There are also distinct functions within nodes of the FPN, where some of the “manipulation” sub-processes of working memory are mediated by parietal cortex, for example, including representations and operations relevant for mental arithmetic [76] and mental rotation [77]. By combining fMRI methods with the measurement of evoked response potentials (ERP), it is possible to track the time-course of FPN control processes: Frontal dipoles contributed prior to parietal dipoles in a task involving updating working memory to bias processing of stimulus-response mappings mediated by the parietal cortex [78].

Theories relating basic processing of external stimulus features for immediate action to future planning functions involving working memory for sequential actions or branching rule contingencies have suggested a linear hierarchy of control operations in the FPN, with the most abstract levels represented by the most anterior PFC structures, i.e., in fronto-polar regions [67, 79]. This hierarchical scheme, supported by evidence from functional neuroimaging and dynamic causal modeling, postulates the caudal lateral PFC to be responsive to external stimulus features, the mid-lateral PFC to contextual rules for attention, and the most rostral parts of lateral PFC to implement rules from working memory. However, causal analyses employing theta-burst transcranial magnetic stimulation (TMS) to reduce cortical excitability of infra-PFC connections have suggested that the “hub” or “apex” where these control influences over attention to the present, external world and the future, “internal” one, are integrated in mid-lateral, not rostral PFC [80].

With respect to *cognitive flexibility*, one of the classical tests in humans, the Wisconsin Card Sort Task (WCST; see Fig. 1), originally implicated both OFC and dlPFC [81], on the basis of lesion studies in humans and monkeys. A modern lesion study [82] in the marmoset showed that excitotoxic, cell body (i.e., fiber-sparing) lesions to the OFC (BA-13) and to the lateral PFC, BA-12/47) produced a clear double dissociation between two distinct tests commonly associated with cognitive flexibility (or inhibitory control): Reversal learning was robustly impaired by the OFC lesion (BA-11), and extra-dimensional set-shifting (as occurs in the WCST) by ventrolateral (vl)PFC (BA-12/47) lesions. (An apparently similar neural dissociation of these deficits has subsequently also been shown in both rats and mice, as well as in humans [83]). Moreover, other studies revealed that the set-shifting deficit occurred in the absence of any obvious “on-line” working memory impairments [84].

These studies suggest dissociation, not only between elements of CC, but also even within the domain of cognitive flexibility. The findings are also compatible with the hypothetically hierarchical organization of PFC function by which reversing contingencies for objects based on changes in value are at a lower level than flexibly attending to perceptual dimensions or categories. There is also evident task impurity in the former case; impairments in reversal learning could have resulted from deficits in the processing of negative feedback or the value of objects rather than cognitive flexibility per se.

Functional neuroimaging studies in humans confirm that these two tests of cognitive flexibility implicate different PFC regions, lateral PFC in the case of set-shifting (compared with intra-dimensional shifts) and OFC regions in the case of reversal learning occurring after negative feedback [85]. Additional analyses in that study suggested that the parietal activations occurred when previous stimulus-reward mappings needed to be overwritten and dlPFC activation at all phases of the task involving new solutions, thus providing a fractionation of the neural regions implicated in attentional control. Another, resting state, study of patients with OCD and healthy controls showed that the ability to perform the extra-dimensional shift task was related to functional connectivity between the ventrolateral PFC and the caudate nucleus, whereas performance of a visuospatial planning task implicated activity in a distinct fronto-striatal pathway [86]).

Whereas set-shifting and reversal learning may depend on learning from reinforcing feedback, switching rapidly between established stimulus-response mappings or task sets may produce switch costs. The latter are caused by the required reconfiguration of task sets and interference between them, and exaggerated by damage to PFC regions, especially to the right and left inferior frontal cortex [87]. Functional neuroimaging studies with several forms of task set switching that isolated perceptual versus response-related aspects of switching highlighted the right inferior junction and posterior parietal cortex as domain general zones for switching [88, 89]. dlPFC was more implicated specifically in response switching, and posterior frontal regions (e.g., premotor cortex) in perceptual switching, with a familiar caudal-rostral PFC gradient of increasingly abstract switching rules [89]. Thus, mechanisms underlying cognitive flexibility overlap anatomically with those of updating working memory to some extent, but likely also depend on some specialized circuitry [90].

To examine *response inhibition*, Aron et al. [91] applied the lesion approach method to a single paradigm, the stop-signal task [92], in group of patients with variable volumes of damage to different sectors of the PFC. They showed that the only sector correlating with stop-signal reaction time was the right inferior frontal gyrus (RIFG; including BA-44 and BA-45, especially pars opercularis). Go reaction time, for example, was more related to damage to other PFC regions. This result was of theoretical significance, as the stop-signal task can readily be considered to measure response inhibition, although, like virtually all other tests of CC, it is impure and also incorporates attentional components. A good deal of evidence from a variety of methodologies, including fMRI and disruptive TMS, has supported this general correlation of RIFG dysfunction with impaired response inhibition [93], but the question has remained whether this region specifically mediates response inhibition or other aspects of performance (e.g., [94–97]). It is notable, for example, that patients with lesions in this area also exhibit deficits in spatial working memory performance [98], consistent with the MD model of lateral PFC function.

The issue of anatomical specificity matching cognitive specificity for stopping inhibition has been addressed from a variety of perspectives. A meta-analysis of the large number of relevant fMRI studies has shown two main peak BOLD activations, one within the insular cortex, apparently coincident with initial processing of the STOP signal, and a subsequent peak focused more in the RIFG, and plausibly associated with the production of the response. A possible solution of the issue may depend on sophisticated network analyses [99]. Early on, Aron and Poldrack [100] proposed a specific circuit for response braking that included the hyperdirect projection from RIFG to the subthalamic nucleus (STN), so it could perhaps be argued that among its various functions, via part of its extensive pattern of connections, the “hub” that is the RIFG has specific “spokes” that mediate relatively specific components of CC. An analysis of effective connectivity among PFC regions during stop-signal task performance revealed that the best selected Bayesian model allowed the RIFG to modulate an excitatory influence of the pre-SMA on the STN, thereby amplifying downstream polysynaptic inhibition from the STN to the motor cortex. Diffusion tensor imaging of the white fiber connectivity of these structures validated these conclusions and predicted individual differences in stopping efficiency [101]. To show CC specificity would require double dissociations of the dynamics of network action of this type, but this analysis clearly identifies highly specific interactions among frontal regions and an important modulatory role for the RIFG “hub.”

In the stop-signal task there is also a right frontal electrophysiological signature of increased beta power for successful versus stop trials, which is matched by a similar signal during a requirement to stop an unwanted thought coming to mind [102]. This match raises the possibility that the right lateral frontal cortex controls a general inhibitory mechanism that does not simply brake actions, but can also inhibit cognitive and emotional outputs [103]. The latter study [103] found that the right medial gyrus contained three nodes of activation that mediated cognitive and emotional inhibitory effects at the more anterior sites and motor inhibition more posteriorly, interacting with the RIFG. The emotional inhibition task engaged the OFC and amygdala, whereas a think-no-think task involved the hippocampus, congruent with the work of Anderson [104, 105], who has consistently shown that memory retrieval can be inhibited by a dlPFC pathway to the hippocampus, via relays in the mid-temporal lobe or retrosplenial cortex (see Anderson & Floresco, this issue [106]). Overall, there is increasing evidence for parallel, top-down inhibitory systems over a range of behavioral and cognitive responses, somewhat lateralised to the right hemisphere. The reasons for this lateralization are not currently clear, but could relate to the lateralization of language to the left hemisphere, or possibly to lateralization of some emotional functions to the right. Existing evidence suggests that the complementary left inferior frontal cortex regions play a role in semantic memory retrieval [107], as well as constituting Broca’s area (BA-44/45).

In the case of *motivational control (“hot” vs “cool” CC)*, perhaps the most striking dissociations for human patients with frontal lobe injury were cases of everyday decision-making in the absence of obvious impairment in IQ or conventional neuropsychological testing of classical “frontal” deficits [108]. Subsequent work established that such deficits were caused by extensive damage to the orbitofrontal and ventromedial (vm)PFC, extending into the frontal pole (BA-10) [109, 110]. Such patterns may indicate a clear division between “hot” and “cool” cognition, the latter comprising what could be termed as CC. By contrast, the vmPFC, an often ill-defined area potentially comprising several distinct cytoarchitectonic regions (Fig. 2), is commonly associated with a “goal-directed” system [7].

Although working memory components incorporate such notions as “goal maintenance,” it is important to consider how PFC circuits mediate the associative learning and monitoring of instrumental behavior, leading to such goals or response outcomes, including their valuation. Such motivational and evaluation functions are the province of PFC regions including the OFC (BA 11,12,13,14) and ACC (BA 24 and 32) ([7]; see also reviews by Monosov & Rushworth, this issue, and Rudebek & Izquierdo, this issue [111, 112]), as well as the neuromodulatory influences of the ascending monoaminergic systems (Cools & Arnsten, this issue [113]).

Early notions of a role for the ACC in inhibition of prepotent dispositions and error monitoring in CC theory have more recently been supplanted by considerations of effort, choice difficulty, and adaptive coding of response outcomes, relevant to flexible foraging behavior and the exploration of alternative choices [114]. ACC activity is enhanced under conditions not only of conflict, but also cognitive effort and choice difficulty [115]. Moreover, studies using fMRI and electrophysiological (error-related negativity) methods in humans, as well as single-unit electrophysiology in experimental animals, have identified the ACC to be a site of computation of prediction errors encoding the difference between expected and obtained outcomes of responding [115–117].

It has proven to be a difficult task to accommodate all of these empirical phenomena in one computational model; how therefore can such a diversity of functions most parsimoniously be explained? Notable have been attempts to incorporate effortful and cost-benefit factors, as in the Expected Value of Control model [118] and the extension of the conflict monitoring notion to decision-making between options of similar value in the Choice Difficulty model [119] (see also Collins & Shenhav, this issue [120]). By contrast, the Predicted Response Outcome model [117] uses as its basis unsigned prediction errors of any type, whether appetitive or aversive outcomes, and whether negative (unexpected omission of expected outcome) or positive (unexpected occurrence of outcome) “surprise,” over multiple time-scales to directly affect actions (and not stimulus representations). This model thus accommodates observations of multiple signals in this region concerning outcomes of actions based on appetitive reward as well as aversive pain [114] and explains non-prepotent responses on the Stroop and errors as being less expected events. Further extensions to the model explain how this information can lead to behavioral adaptation during decision-making and to both proactive and reactive CC [121], in terms of anticipatory adjustments to responding on one hand, as in risk-avoidance and foraging behavior, and to error-induced slowing caused by negative surprise and the temporary invigoration of responding (“hot-hand”) of repeated positive surprise in association with rewarding feedback [122] on the other. The latter computational model has recently been suggested to capture the most important functions of the entire PFC [122].

A recent fMRI test of speeded value-based decision-making that could distinguish amongst the predictions made by the various models has favored the Predicted Response Outcome model over the Expected Value of Control and Choice Difficulty alternatives [123]. However, the Predicted Response Outcome model has a characteristic signature in several other brain regions, including the lateral PFC and parietal cortex, that pose questions about how signals from the ACC are relayed to other regions of the brain, including to the fronto-parietal axis, the striosomes of the striatum, and the noradrenergic locus coeruleus, with diverse applications. Moreover, it is possible that the differing strengths of the models may be reflected by anatomical differentiation within what is a large, quite heterogeneous anatomical region. Thus, choice difficulty has been related to a more dorsomedial region close to the pre-supplementary motor area and pain to more ventral regions of the ACC [114, 122].

### Neurochemical modulation of CC

CC has to occur in the context and history of different motivational states including those produced by prior stress and learning, mediated in part by phasic and tonic changes in the ascending monoaminergic and cholinergic neurotransmitter systems. For example, dopamine receptors in the PFC have been related hypothetically to three main elements of CC: gating; maintaining and relaying motor commands; and producing error learning signals [116, 124, 125].

An extensive literature in human and experimental animals has shown that CC is susceptible to pharmacological intervention and hence to neuromodulation by the ascending monoaminergic and cholinergic systems [126, 127] (see also Cools & Arnsten, this issue [113]). For example, catecholaminergic drugs such as methylphenidate and atomoxetine, or manipulations affecting the dopaminergic and noradrenergic neurotransmitter systems, can affect several aspects of CC, including working memory, cognitive flexibility, and response inhibition. However, drug effects are generally dose-dependent and conform to a familiar inverted U-shaped function. Moreover, different tasks reflecting different components of CC may be affected in dissociable ways. For example, dopamine depletion in the marmoset PFC impaired spatial delayed response whilst enhancing extra-dimensional shifting [128]. Floresco [129] reviews data suggesting that dopamine D1 receptors are more implicated in working memory, whereas D2 receptors promote cognitive flexibility in rodents. In patients with Parkinson’s disease, therapeutic doses of L-Dopa appear to improve working memory and task-set switching but impair reversal learning and decision-making [130].

On the other hand, serotonin depletion of the OFC in marmosets impaired reversal learning without affecting extra-dimensional set-shifting [131]. In human studies, the noradrenergic reuptake inhibitor atomoxetine enhanced stop-signal performance but had no effect on probabilistic learning known to engage OFC mechanisms, whereas the selective serotonin reuptake inhibitor citalopram had the reverse pattern of effects [132]. These findings indicate a degree of specificity in how these ascending transmitter systems interact with PFC–striatal networks and also raise the possibility that the motivational states mediated by activity in these systems may differentially prime different aspects of CC. The possibility of “top-down” or local control of the cholinergic system [133] or the catecholamine system [134] by PFC circuitry may also provide mechanisms for the allocation of “cognitive effort”.

### Integration of CC

Despite the evidence for fractionation (“diversity”) of CC, there is also considerable evidence for overlap (“unity”) across separable CC/EF processes. As described earlier, at the behavioral level, this unity can be observed in the correlations among EF latent factors, which enables estimation of a “Common” CC factor (Fig. 3). At the neurobiological level, this unity can be seen in the overall patterns of neural activations during CC tasks [135–137]: Neuroimaging studies suggest that individuals performing these kinds of tasks generally activate some of the same brain regions across multiple tasks, although they also activate some regions that are unique to a particular task.

Conjunction maps of activation during different tasks (e.g., during inhibiting, updating, and shifting tasks), either with data estimated within one study [135, 138, 139] or with meta-analytic techniques [137, 140, 141], suggest that both children and adults recruit a common set of regions within the FPN and CON during diverse EF tasks. The FPN and CON are functionally separable brain networks that enable flexible adaptive control and sustained task-set maintenance [142]; together, they form the MD network [143], a set of brain regions that is active during a range of tasks that require goal-directed behavior [139]. For example, Niendam et al.‘s [137] meta-analytic study of 193 neuroimaging fMRI studies of EF showed broad patterns of activation across the lateral and medial PFC, including regions BA-9 and BA-46 (dlPFC) and BA-32 (rostral ACC), as well as both superior and inferior regions of the parietal lobes for tasks with predominantly working memory, flexibility, or inhibitory components.

However, despite overall conjunctions of activation, there may be subtle differences among tasks that also account for diversity. In Niendam et al.‘s [137] meta-analysis, flexibility tasks alone activated BA 11 and a sub-class of tasks involving initiation did not activate parietal regions. A similar conclusion was arrived at by Fedorenko et al. [139] using a more refined design by which individual subjects were shown to exhibit overlapping activation in a variety of tasks including verbal and spatial working memory, arithmetic, Stroop, and attentional tasks. The concept of diversity as well as unity of PFC functions can be illustrated in a typical fMRI study [144] which had defined task components of response inhibition and cognitive flexibility, finding increased BOLD activity in the FPN including both the RIFG region and in the parietal cortex. However, whereas the response control-related activity was greatest in the RIFG, the opposite was the case for attentional shifting; hence the different nodes of the FPN as well as functioning as part of a network, evidently had different roles within that network. The FPN can be regarded in some sense as a domain-general system that flexibly connects with other networks depending on the nature of the task, but the various dissociations reviewed also highlight how CC can be deployed in specific ways, perhaps capturing diversity as well as unity of PFC function.

Although there are clearly common neural areas recruited by diverse CC tasks, an open question is whether these same areas are involved in individual differences in performance of these tasks. That is, most people activate the FPN during demanding tasks, but do performance differences across tasks systematically relate to how strongly individuals activate the FPN or its nodes during these tasks? Few studies have looked at conjunction maps of areas related to individual differences in performance, but one study that did so did not find areas of significant overlap across three CC tasks, despite significant areas of mean activation across those tasks [138].

Studies that have correlated individual differences in Common CC ability with a neural measure, such as functional activation of a particular region during a task, functional or structural connectivity, or gray matter volume, often find that areas within but also outside of the MD network contribute to performance [145–148]. A meta-analysis of healthy adults [149] found that better performance on individual CC tasks was associated with larger PFC volume and greater PFC thickness, particularly for lateral PFC, although there was significant heterogeneity in the strength of association that was related to the particular tasks examined as well as sample age variability. Examining a group of healthy college-aged young adults, Smolker et al. [147] found that a Common CC factor was related to changes in gray matter and cortical folding in the broad vmPFC area, typically associated with the goal-directed system, whereas the specific aspects of updating of working memory and cognitive shifting were related specifically to dlPFC and vlPFC, respectively. However, it is notable that using a latent factor analysis in concert with structural imaging, there was only limited evidence of a major involvement of fronto-parietal activity for the Common CC factor.

A later study [148] in a large group (*N* = 251) of healthy adults approximately a decade older, and employing a larger test battery, did not confirm all of these associations, possibly because of an important developmental factor. In this study, Common CC was related to greater volume of the right middle frontal gyrus/frontal pole and to fractional anisotropy of the right superior longitudinal fasciculus, connecting the frontal lobes to other regions of posterior neocortex, including the parietal lobes. Updating-specific ability was linked to gray matter changes in regions both within and outside the fronto-parietal axis. In contrast, Shifting-specific ability implicated widespread white matter changes, as measured by mean, radial and axial diffusivity measures. In a similarly aged sample (ages 22–35) from the Human Connectome Project, Lerman-Sinkoff et al. [150] examined correlates of a Common CC composite using a data-driven approach (independent components analysis) to reduce multimodal imaging data. They found that CC was related to two components, one of which included variation related to visual network activity and insular gray matter volume, and the other of which included activity of the FPN and gray matter thickness in the CON [150].

Taken together, these results suggest that individual differences in Common CC are linked to structural and functional characteristics of the brain that include the FPN and CON, but also networks related to lower-level processes such as the visual network. Stronger global or specific connectivity of the dlPFC to other regions throughout the brain has been associated with better performance on diverse CC tasks or factors [151, 152]. As such, dlPFC seems to affect individual differences in performance through its influence on other areas within and outside of the FPN.

Finally, a number of recent studies that have examined variations in structural and functional connectivity also suggest that more large-scale properties (as opposed to region-specific or even network-specific properties) may relate to Common CC. A large study of participants aged 8–22 years [153] found that higher scores on a CC factor were related to higher modularity of white matter brain networks (stronger connections within networks and weaker connections between networks), particularly the FPN, and modularity mediated age-related increases in CC scores. More modular, segregated networks may allow for more specialization and less interference across brain networks. CC scores were also positively associated with global efficiency, a measure of how quickly information can flow across networks. These results suggest that higher Common CC is associated with brain structures characterized by both more specialization of networks but also greater coordination across networks. Moreover, the extent to which brain network connectivity changes in response to cognitive demands is associated with better performance on different CC tasks [154, 155], suggesting that task-based neural flexibility may facilitate Common CC through adaptive control [156].

The robust evidence for shared variance and common patterns of neural activations across CC tasks begs the key question of what cognitive process(es) comprise the “unity” of CC. There are a number of proposed mechanisms for the Common CC factor, which have both informed and been informed by the neuropsychological literature.

One proposed mechanism for the Common CC factor is *inhibition*, a mechanism apparently consistent with a strong relationship between the Common CC factor and the response inhibition factor that has been observed in several studies: When a higher-order common factor is used to model the correlations among response inhibition, working memory updating, and mental set shifting (see Fig. 3b), that common factor almost perfectly predicts the inhibition factor [37, 38, 157, 158], but there is significant variance in the updating and shifting factors that is not related to the common factor. Similarly, when the correlated factors model is re-parameterized into a bifactor model (see Fig. 3c), there are updating-specific and shifting-specific factors, but there is no evidence for a response inhibition-specific factor [37, 38, 157]. In other words, the variance that is shared across response inhibition tasks is the same variance shared across all tasks (response inhibition, working memory updating, and mental set shifting). Broadly speaking, this pattern suggests that, at the level of individual differences, unity (what is common to CC processes) may be isomorphic with response inhibition, whereas diversity is evident in additional processes associated with updating working memory and mental set shifting.

This pattern of little inhibition-specific variance could be interpreted as indicating that what is common to CC abilities *is* inhibition. That is, one could describe most if not all CC processes as requiring some sort of inhibition [159]. For example, updating working memory tasks could be characterized as requiring inhibition both to stop irrelevant information from entering working memory and to remove no-longer-relevant information from working memory when appropriate [160]. Similarly, mental set shifting tasks could be characterized as requiring inhibition to ignore information irrelevant to the current task set [161], as well as to suppress the no-longer-relevant task set when switching sets [162].

However, this characterization relies on the assumption that processes described with similar terms (such as “inhibition”) are in fact similar, an assumption that may not be valid [163]. Even though the same inhibition term is used to describe these requirements, those processes may be dissociable [161, 164], and in some cases, may not involve inhibition (i.e., neural inhibition) at all [165, 166].

An alternative proposal is that the unity component reflects individual differences in the ability to *actively maintain goals and use those goals to bias ongoing processing* [29, 97, 167]. To perform well in all CC tasks, participants must have accurate representations of the task goals that can be used to direct attention to task-relevant information, particularly when there is conflicting task-irrelevant information. In some cases, participants must also monitor the environment for conditions that signal that the goal is relevant. For example, in stop-signal tasks, the stop goal is only relevant on a subset of trials in which a signal occurs, so performance may partially depend on being able to quickly recognize the relevance of the signal and stop the response [95]. According to this proposal, *individual differences* in response inhibition tasks may be particularly related to this ability because if a goal is inactive or ineffective, then more automatic or prepotent responses will take over, leading to poor performance on these tasks. If response-inhibition-specific neural processes, such as global motor suppression [93, 168], do not show large individual differences, then task performance may be more driven by whether those processes are triggered in the first place. To the extent that keeping goals highly active and proactively biasing ongoing processing influences stopping, performance may be more determined by these global processes rather than inhibition-specific ones [29, 97, 169].

This proposal that unity captures goal maintenance and biasing [29] is in fact a classic conception of CC and frontal lobe function [8, 170, 171]. In addition to proposing that CC involves the active maintenance of goals in the PFC that bias processing elsewhere in the brain, Miller and Cohen [8] argued that this key function of PFC is responsible for various aspects of CC, such as selective attention, response inhibition, and working memory. Seen from an individual differences perspective, they essentially proposed a “common” CC ability that depended on such goal maintenance and bias.

Specifically, building on Desimone and Duncan’s [170] model of such competitive dynamics during visual attention, Miller and Cohen argued that prefrontal goal representations enable weak stimulus-response mappings to out-compete more habitual ones when appropriate: Goal representations bias competition by boosting activation for task-relevant processing, which, by virtue of lateral inhibition, suppress activity of competing representations. In this sense, “inhibition” could be seen as fundamental to common CC ability. Yet, this goal maintenance and biasing account of common CC ability is conceptually different from accounts that invoke a broader inhibition mechanism, discussed earlier.

The goal maintenance/biasing perspective is incorporated into several other CC frameworks, such as the executive attention framework [172] and the dual mechanisms of CC framework [121]. Duncan and colleagues’ MD framework also prominently incorporates a goal-maintenance/biasing perspective. They characterized the unity of frontal lobe functions in terms of goal-related processes, specifically the ability to form and carry out goals at multiple levels of abstraction [32]. Failures in this ability can manifest as goal neglect, a phenomenon commonly observed with head injury, as also noted by Teuber [31]. Duncan et al. [32] found that goal neglect was more related to general brain atrophy than focal frontal lesions, and Duncan [11] later linked these general goal-related processes to the MD network a network of frontal and parietal regions that is commonly activated across tasks.

Duncan and colleagues [11, 32] have also linked goal neglect and the activity of the MD network with general fluid intelligence, leading to the last interpretation of the unity component we consider here: that it recapitulates *intelligence or Spearman’s g*. Indeed, a large body of work has documented moderate to large correlations between intelligence measures, particularly measures of fluid intelligence (such as reasoning) with measures of CC. Perhaps most relevant, studies that have measured a common CC latent factor have reported correlations with intelligence ranging from *r* = 0.53–0.91 [41, 158, 173, 174]. Such correlations suggest that the unity of CC is related to intelligence or *g*. However, at least in adult samples, this correlation is only moderate, and is significantly lower than 1 (*r* = 0.53–0.68 [41, 158]), indicating that these constructs cannot be considered identical, even when examined with latent variables. Moreover, CC and intelligence seem to show discriminant predictive validity of behavior, in that CC is associated with problems related to attention-deficit/hyperactivity disorder (e.g., [175, 176]) or lack of self-restraint [177], even when controlling for intelligence.

In addition to correlating with common CC, intelligence also significantly correlates with the variance that is unique to working memory processes (working memory updating and/or capacity) [41, 158]. Such results suggest that although the CC unity component may reflect *some* of the same processes tapped by intelligence measures, common CC is not equivalent to intelligence. Rather, intelligence may be related to both common CC and working memory-specific processes, consistent with earlier research showing that intelligence and particularly reasoning ability are strongly related to working memory capacity [178, 179].

## Clinical implications

Many psychiatric and neurological disorders are associated with specific symptoms that may be at least partly a product of impaired CC, or with more general cognitive deficits that accompany the specific symptoms. For example, Attention-Deficit Hyperactivity Disorder (ADHD) has major EF/CC impairments in attentional control, working memory, and response inhibition that contribute to DSM-5 symptoms of distractibility and impulsivity. Similarly, some symptoms of major depressive disorder include problems of decision-making and concentration, which appear to entail primary CC impairments. In schizophrenia, negative symptoms have been related to impaired goal-directed behavior [180] and positive symptoms such as delusions and hallucinations to deficits in reality monitoring [181], although there is an additional domain of symptoms in schizophrenia of cognitive impairment that includes major working memory deficits and impedes rehabilitation [182].

One hypothesis concerning addiction is that it results from a general impairment in goal-directed behavior, leading to more pronounced habitual tendencies, and exacerbated by a loss of top-down control, to contribute to compulsive drug-seeking [183]. An analogous mechanism has been proposed to account for other forms of compulsive behavior, such as checking or washing in obsessive-compulsive disorder [184]. However, it should also be recognized that hyperactivity in medial PFC regions in such disorders (Ahmari & Rauch, this issue [185]) could potentially be associated with specific compulsive behaviors that retain their goal-directness. Moreover, it is possible that a simple dichotomy between goal-directed and habitual behavior is too simple. A recent computational formulation [186] has suggested an intermediate type of control mode relying on model-based and model-free computations guided by “successor representations’“ that enable behavior to be both flexibly goal-directed but also efficiently model free.

Although the precise contribution of dysfunctional CC mechanisms to psychiatric and neurological symptoms, perhaps in combination with altered perceptual and motivational processes, remains to be determined, at a general level, it is clear that CC deficits are characteristic of a wide range of disorders. Indeed, meta-analyses suggest transdiagnostic associations of CC deficits with psychiatric disorders, including major depressive disorder, post-traumatic stress disorder [187], obsessive-compulsive disorder [188], bipolar disorder [189], schizophrenia [190], ADHD [191, 192], conduct disorder and antisocial personality disorder [193], and substance use disorders [194].

### Unity and diversity of psychopathology in relation to CC

Although such psychiatric and neurological disorders are often treated as distinct entities, a growing body of work has focused on the observation that these disorders share considerable variance [195]. That is, whether treated as dimensional or categorical constructs, different disorders are often comorbid, either concurrently or sequentially across the lifespan [20, 195]. This common variance occurs at multiple levels of specificity. At one level, particular disorders can be clustered into internalizing (depression and anxiety), externalizing (antisocial behavior and substance use), and thought disorder (schizophrenia, bipolar disorder, obsessive-compulsive disorder) factors. At a higher-order level, these internalizing, externalizing, and thought disorder factors correlate with each other, and these correlations can be modeled with a general psychopathology factor [20, 195, 196]. This hierarchical general factor has been dubbed the “*p* factor” in recognition of its parallel to the *g* factor for cognitive abilities [197]. The *p* factor has been modeled in a number of datasets, and shows longitudinal stability and criterion validity, in that it predicts a number of clinical outcomes [195, 196].

However, like any statistical factor, it describes a pattern of correlations but not an explanation of those correlations. That is, its neurobiological underpinnings are not well understood and its psychological interpretation varies [20, 195]. For example, the *p* factor has been proposed to reflect negative emotionality, disordered thinking, and/or poor CC, particularly impulse control (inhibitory control) over positive and negative emotions [2, 20, 195, 198].

With respect to CC, the transdiagnostic associations of CC with psychopathology support the notion that the *p* factor may partly reflect CC deficits [1, 2, 197], although there may also be specific CC deficits associated with particular disorders or clusters of disorders (e.g., [197, 199]). Moreover, within many disorders, it appears that multiple aspects of CC (inhibition, shifting or flexibility, and working memory processes) are impaired [1], though possibly to different extents. These patterns suggest that CC impairments associated with psychopathology may be general, reflecting variance that is shared across multiple CC constructs [1]. Indeed, several studies have examined this hypothesis directly, finding that a common CC factor is associated with a *p* factor (*r* = –0.16 to –0.56) [174, 200–203].

Studies of neural correlates of psychopathology also suggest the importance of CC-related regions of interest and networks. In particular, multiple psychiatric disorders are associated with hypoactivation of the FPN and CON during CC tasks [2, 204], alterations in functional connectivity of these networks at rest [3, 205], and alterations in gray matter volume in nodes of these networks, including the dorsal ACC, insula, and dorsomedial and vmPFC [2, 3, 206]. When integrated with findings that these same patterns are associated with poorer performance on CC tasks [2, 3], these results are consistent with the conclusion that CC and mental health share neural substrates, and that disruptions of these neural substrates may account for increased *p* and decreased common CC functioning [2, 3].

### Links between impulse control, CC, and psychopathology

Many PFC areas are particularly associated with control over emotionally relevant information (hot CC) [60–64, 66], which, as discussed earlier, show some dissociations from cool CC. These associations are consistent with interpretations of the *p* factor that focus on emotional regulation, particularly impulse control in the context of high arousal (both positive and negative emotion) [198]. At the behavioral level, such emotional impulse control is often measured with self-report measures of impulsivity and emotional urgency, such as those assessed with the UPPS-P impulsivity scale [207].

Although such emotional impulse control is thought to be enabled by general processes and neural correlates of CC [198], and urgency measures are correlated with CC, these correlations are generally weak (*r* = ~0.10 to 0.20), as are correlations between laboratory CC tasks and more general self-control and EF questionnaires [157, 208–212]. These weak relationships could indicate that there is no great dependency of impulse control on CC processes or the PFC, or indicate that subjective report of impulse control represents a domain of EF outside classical CC function, or it could simply reflect methodological differences. For example, the self-report questionnaires measure subjective aspects of performance whereas laboratory tests such as the Stroop measure objective aspects. However, some evidence suggests that task-based and self-report measures of CC may best be considered separable constructs that are both relevant to mental health [209, 213], because they independently predict psychopathology in multiple regressions [157, 214, 215]. This conclusion is consistent with the possibility that these different aspects of CC may depend on different PFC regions: e.g., self-report measures have been correlated with medial PFC morphology, whereas CC tasks typically activate more lateral PFC [216].

Whether other-dimensional measures of performance, such as apathy (e.g., as measured by the Apathy Motivation Index [217]) or compulsivity (as measured by the Obsessive-Compulsive Inventory, OCI [218]) will be beset by similar issues is as yet unclear. One potentially important approach has been to combine computational paradigms such as the two-stage Markov decision-making task with latent factors including compulsivity from an analysis of multiple questionnaires used in impulsive-compulsive disorders [219]. This study showed that a factor of compulsivity was related to a bias to “model-free” responding, over “model-based” responding, which is commonly associated with goal-directed behavior. Participants in the “model-free” mode tend to respond according to the “win-stay/lose-shift” heuristics of Thorndike’s Law of Effect underlying reinforcement learning, whereas “model-based” responding entails developing a “mental model” of the task, which may involve higher-order processes of CC to optimize performance (e.g., switching away from win-stay when it is ultimately advantageous to do so).

### Causal direction of associations between CC and psychopathology and substance use

Although it is clear that CC deficits are behaviorally associated with psychopathology, the causal origin of these relationships are often unclear. Are CC deficits a cause or consequence of emotional and behavioral problems, or perhaps both (i.e., is there a bidirectional relationship)? And if CC deficits are a consequence of the psychopathology, do they produce exacerbation of those symptoms, or other distinct problems that require rehabilitation? An obvious example is substance use disorders, where pre-existing deficits in CC may predispose to drug taking, but drug taking may also cause CC deficits by producing neuropathology, for example in the PFC and related circuitry. Cause-effect relationships regarding other forms of psychiatric morbidity can plausibly operate in a similar fashion. However, it is also possible that these relationships reflect common associations with other variables (e.g., correlated genetic or environmental risk factors).

Quasi-experimental observational designs such as family studies provide some evidence that these associations are at least partly due to correlated genetic risk. For example, stimulant drug abusers and their first degree relatives both have deficits in response inhibition on the Stop task, correlated with reductions in white matter in the RIFG [220]. Whilst this can be interpreted as showing that PFC-related response inhibition deficits promote vulnerability to stimulants, this influence of impaired response inhibition could theoretically arise from family-related environmental, as well as genetic, influences. Twin studies suggest that associations between psychopathology/substance use and CC are attributable largely to shared genetic influences [157, 221]. However, there is some evidence for correlated environmental influences in addition to correlated genetic influences for a common CC factor with depression symptoms in a middle-aged male twin sample [222] and for a common CC factor with a *p* factor in children and adolescents [200].

Several co-twin control studies, which examine relationships controlling for shared familial risk factors, generally suggest that associations of lower cognitive ability with substance use, particularly cannabis, are not consistent with causal models in which substance use causes cognitive impairment. Specifically, the twin who used cannabis more often or began using earlier did not have lower cognitive ability or brain volume than their co-twins, which is inconsistent with a causal effect of the drug [223–226]. However, a recent co-twin-control study of young adults [227] found that the association of alcohol, but not cannabis, misuse with reduced cortical thickness of central executive and salience networks was consistent with causal effects of alcohol exposure as well as pre-existing genetic associations of cortical thickness with the propensity to misuse alcohol. Specifically, causal effects of alcohol misuse were present for lateral PFC, medial frontal and parietal areas, and the frontal operculum (BA 44). These results are thus consistent with a model in which reduced cortical thickness in areas that enable CC, particularly those related to response inhibition, may increase risk for alcohol misuse, and subsequent misuse further impacts those cortical areas (see also [228]).

## Summary and future research directions

Lesion studies and psychometric models both suggest unity and diversity of CC. CC tasks that assess processes such as response inhibition, interference control, working memory maintenance and updating, and mental set shifting show unique variances, but also exhibit some overlap. This overlap (the “unity” of CC) can be characterized in multiple ways, but most characterizations include goal-related processes, such as active goal maintenance and the use of such goals to bias ongoing processing. It appears that there are CC processes that distinguish working memory updating, mental set shifting, and potentially other functions (dual-tasking ability and generativity, as in verbal fluency) from the common CC factor. It is also clear that hot CC can be distinguished from cool CC, and that CC as measured by laboratory tasks is quite different from constructs like impulsivity, which are typically measured with self-reports but can also be measured with laboratory paradigms. Given that different “objective” measures of impulsivity often fail to inter-correlate themselves [229], and there is also neural evidence of dissociation [230], it is likely that impulsivity, like CC and inhibition, is a multi-dimensional construct that includes a family of related but separable processes and underlying neural systems. Both CC and self-reported dimensions such as impulsivity may independently relate to psychiatric dysfunction, perhaps at different levels (i.e., at the level of individual disorders or factors that capture variance common across disorders).

Our understanding of the “unity and diversity” of PFC function at the neural level is necessarily incomplete, but suggests some congruency with the evidence at psychometric levels. There is evidence, for example, that networks involving the PFC, for example, the FPN, can mediate superficially different types of cognitive performance, suggesting the operation of an MD system of CC. Nevertheless, the existence of functional dissociations following different types of intervention is also compelling and may suggest that there is specialization of circuitry conferred by the flexible networking of its “hubs” with other neural circuitry. In particular, different PFC nodes within the network, as well their interactions with other neural circuitry, presumably have distinct contributions to information processing, and elucidation of such dynamic transactions in real time will be an important future focus of research. Finally, it appears likely that CC will have to be understood in the context of complementary motivational control networks, including subcortical influences of chemical neuromodulatory systems. Thus, the heterogeneous, but also overlapping, nature of psychiatric symptoms across different DSM5 categories presumably reflects the unity and diversity of CC.

The PFC and its associated networks will thus continue to be a major factor in understanding psychiatric and neurological disorders and developing new treatments. We can foresee future research priorities in several areas. The unity and diversity model of CC/EF needs to be developed further to explore other possible constructs related to PFC networks, perhaps especially hot CC, which may be of greatest significance to mental health disorders. It may also prove necessary to decompose some of its existing constructs, e.g., working memory updating and cognitive flexibility (e.g., set shifting), into their components in order to relate them to distinctive psychiatric symptoms and neural dysfunction.

There is also a need to compare different theoretical positions, such as the cognitive, learning theory, and computational modeling approaches, to optimize our descriptions of phenotypes for mapping onto PFC networks. Such refinements could perhaps enhance genetic studies, as well as improve new nosological systems such as United States’ National Institute of Mental Health’s Research Domain Criteria (RDoC) [231], a research framework that advocates examining mental disorders from the perspective of basic dimensions of functioning, each examined at multiple levels of analysis (genes to circuits to behavior) that may apply to multiple diagnostic categories. Most relevant to this review, the RDoC includes cognitive systems, with CC and working memory constructs, but their dysfunctioning in mental health disorders has ultimately to be related to their neural substrates and pathophysiology.

Understanding the unity and diversity of genetic influences on CC and how they map onto associated PFC development and structure is another priority for future research. Structurally, there is evidence of differential genetic regulation of different PFC regions; for example, development of the mouse dorsal (and not ventral) PFC is especially sensitive to the fibroblast growth factor family of genes [232]. Several independent twin studies [36, 39, 41, 157, 233] have yielded evidence that at the latent variable level, CC constructs are moderately to highly heritable and, importantly, that the separability of working memory updating and mental set shifting from the common CC factor is largely attributable to *different* genetic influences.

However, the specific genes that account for these patterns, presumably in part via expression in the PFC, have yet to be identified. Most genome-wide association studies (GWAS) to date have focused on intelligence or *g* [234–237], and suggest that hundreds to thousands of genes additively influence variation in intelligence, with the effect of any one gene being very small (typically in GWAS, a variant has *r*^2^ < 0.05% [238]). The largest GWAS of CC to date [239] included individual CC tests such as the Stroop task with samples smaller than 11,000 individuals, and did not yield any significant associations. Clearly, more work is needed with sufficiently large samples to enable GWAS. However, acquiring detailed cognitive task data on such large samples (*N* = 10’s to 100’s of thousands) is no easy feat and will most certainly require harmonization across multiple samples and/or online testing. Though resulting measures are typically crude compared to the measures included in smaller studies [240], the trade-off between phenotype depth and sample size may be effective for gene discovery [241], as demonstrated by a recent preliminary report [242] of a GWAS for the Common CC factor.

Once such variants are identified, bioinformatic follow-up analyses can be used to identify genetic pathways that influence CC-related neural differences. For example, a recent GWAS [243] suggested that global measures of cortical surface area and thickness were related to distinct genetic influences associated with different developmental mechanisms (i.e., associated with regulatory elements present during fetal development and in adults, respectively); and total surface area was bidirectionally causally related to general cognitive ability and educational attainment. Similar analyses applied to more nuanced CC phenotypes could confirm hypothesized pathways and suggest new avenues of research for understanding behavioral and neural variation related to CC variation and associated clinical outcomes.

As developmental studies are also likely to be of increasing importance for determining the factors influencing the etiology of mental disorders, large scale longitudinal studies of CC/EF, combined with sensitive clinical scales, trait questionnaires, neuroimaging, and genotyping, as for example, in the National Institutes’ of Health Adolescent Brain Cognitive Development (ABCD) study [244], will be invaluable. Such an ambitious project may well have to involve increasingly sophisticated ways of obtaining this information via on-line testing.

Deficits in neural networks, including the PFC, are increasingly being used to determine the neural substrates of CC/EF. However, more analysis is required of the underlying pathophysiology of these networks (e.g., at the circuit and molecular levels), because a network abnormality could arise in many different ways that may have significance for diagnosis, drug discovery, and neuromodulation strategies. Finally, if experimental animals are to be used to model genetic and molecular deficits in the developing brain, new research needs to be done to test whether the “unity and diversity” approach applies across species and fits what is known of PFC homology (Preuss & Wise, this issue [245]).
